# Prognostic significance of interleukin 6 serum levels in patients with ovarian cancer.

**DOI:** 10.1038/bjc.1995.71

**Published:** 1995-02

**Authors:** G. Scambia, U. Testa, P. Benedetti Panici, E. Foti, R. Martucci, A. Gadducci, A. Perillo, V. Facchini, C. Peschle, S. Mancuso

**Affiliations:** Department of Gynecology and Obstetrics, Catholic University, Rome, Italy.

## Abstract

High levels of IL-6 were found in 50% of 114 patients with primary ovarian cancer. IL-6 sensitivity was lower than that of CA 125, and the combination of both assays did not increase the sensitivity of CA 125 alone. However, elevated IL-6 serum levels were correlated with a poor prognosis since patients with low IL-6 levels had a better survival than patients with high IL-6 levels (P = 0.0009). Multivariate analysis revealed that IL-6 positivity has an independent value.


British Journal of Cancer (1995) 71, 354-356

© 1995 Stockton Press All rights reserved 0007-0920/95 $9.00

### Prognostic significance of interleukin 6 serum levels in patients with ovarian cancer

G Scambia[1], U Testa[2], P Benedetti Panici[1], E Foti[1], R Martucci[2], A Gadducci[3], A Perillo[1], V Facchini[3], C Peschle[2] and S Mancuso[1]

[1]*Department of Gynecology and Obstetrics, Catholic University, Rome, Italy;* [2]*Department of Hematology and Oncology, Istituto Superiore di Sanità, Rome, Italy;* [3]*Department of Gynecology and Obstetrics, University of Pisa, Pisa, Italy.*

**Summary** High levels of IL-6 were found in 50% of 114 patients with primary ovarian cancer. IL-6 sensitivity was lower than that of CA 125, and the combination of both assays did not increase the sensitivity of CA 125 alone. However, elevated IL-6 serum levels were correlated with a poor prognosis since patients with low IL-6 levels had a better survival than patients with high IL-6 levels ($P = 0.0009$). Multivariate analysis revealed that IL-6 positivity has an independent value.

**Keywords:** interleukin 6; cytokines; ovarian cancer; serum markers

Recent evidence has shown that IL-6, a multifunctional cytokine that regulates immune responses, acute-phase reactions and haematopoiesis, stimulates the proliferation of ovarian cancer cells in culture (Wu *et al.*, 1992). Moreover, it has been reported (Watson *et al.*, 1990; Berek *et al.*, 1991) that ascites of ovarian cancer patients contain high levels of IL-6 which correlate with disease status. Our previous report demonstrated that high serum IL-6 levels are a unique feature of ovarian cancer as compared with other gynaecological malignancies (Scambia *et al.*, 1994) and that, although IL-6 is less sensitive than CA 125 as a tumour marker for ovarian cancer, the production of IL-6 may be related to tumour aggressiveness.

The present study investigated the prognostic significance of serum IL-6 levels in 114 primary ovarian cancer patients. The correlation between IL-6 and the IL-6 soluble receptor (IL-6R) was also investigated.

### Patients and methods

A total of 114 patients with malignant ovarian tumours admitted to the Department of Gynecology of the Catholic University or to the Department of Gynecology of the University of Pisa from June 1987 to December 1993 were enrolled in the study. Seventy-four healthy women aged 22-68 years were included as controls. The World Health Organization (WHO), method of histological typing of ovarian tumour was adopted (Serov *et al.*, 1973).

After surgery patients received cisplatin-containing regimens (Benedetti Panici *et al.*, 1993). Patients who initially had only an explorative laparotomy underwent a second laparotomy after chemotherapy, and a second cytoreduction was attempted. Venous blood samples for marker determinations were separated by centrifugation and aliquots were stored at $-20°C$ until assay. IL-6 and IL-6R assays were performed using commercially available specific enzymatic immunoassays (RandD Systems Minneapolis, MN, USA). The detection threshold for IL-6 evaluation was $0.3 \text{ pg ml}^{-1}$. The intra-assay variability for IL-6 and IL-6R analyses was 5-20% and 5-10% respectively. Since >95% of normal controls exhibited IL-6 serum levels within the range $1.9-6 \text{ pg ml}^{-1}$, $6 \text{ pg ml}^{-1}$ was used as the cut-off value. Normal control values of IL-6R ranged between 14.9 and $46.4 \text{ ng ml}^{-1}$ (mean value $26.5 \text{ ng ml}^{-1}$). CA 125 was measured using a commercially available kit (CIS, Compagnie ORIS Industrie SA) and the upper limit of CA 125 for normal controls was $35 \text{ U ml}^{-1}$ (Scambia *et al.*, 1990).

The chi-square and Fisher's exact test were used to analyse the relationship between IL-6 serum values and tumour characteristics. All medians and life tables were computed using the product-limit estimate by Kaplan and Meier (1958), and the curves were examined by the log-rank test (Mantel, 1966). Multivariate analysis was performed with BMDP statistical software (Dixon, 1981). A backward stepwise procedure was used to identify the major prognostic factors. Survival time was calculated from the date of diagnosis to the date of death.

### Results

Table I shows the comparison of IL-6 distribution and positivity of IL-6 and CA 125 according to clinocopathological parameters. IL-6 positivity was not significantly related to stage, histology, grading or presence of ascites. However, the percentage of IL-6 positivity was significantly higher in patients with post-operative residual tumour >2 cm as compared with patients with residual tumour ⩽2 cm ($P = 0.02$). Although no linear correlation between IL-6 and CA 125 levels was found, the overall sensitivity was only slightly increased by the combination of IL-6 and CA 125. Ninety-two per cent of the serum samples showed a positive reaction in at least one test as compared with 87% for CA-125 alone (data not shown).

Soluble IL-6R was also measured in 44 patients. Receptor levels were significantly increased as compared with normal controls (mean ± s.e.m 127 ± 1.1 range, 109.7-153.1, *vs* 26.5 ± 1.04, 14.9-43.4). No correlation between IL-6R and IL-6 serum levels was found ($P = 0.27$, $P = 0.15$). Moreover, IL-6R was not related to any clinicopathological characteristics (data not shown).

Survival analysis of the overall patient population showed a shorter survival for patients with high IL-6 levels as compared with those patients presenting normal or low IL-6 values ($P = 0.001$). However, in order to avoid possible bias owing to the inclusion of patients with early-stage disease, survival analysis was limited to patients with stage II, III and IV ovarian cancer. Follow-up data were available for 83 of these patients, and death occurred in 38 cases. Figure 1a shows the cumulative percentage survival as related to serum IL-6 status at initial diagnosis. A highly significant correlation between patients with low IL-6 levels and a longer survival compared with patients with high IL-6 levels ($P = 0.0009$) was observed. Median survival was 21 months for patients with high IL-6 serum levels as compared with 51 months for patients with low IL-6 values. Figure 1b shows

Correspondence: Mancuso, Department of Gynecology and Obstetrics, Catholic University, Largo A. Gemelli, 8, 00168, Rome, Italy
Received 29 April 1994; revised 12 August 1994; accepted 7 September 1994

**Table I** IL-6 serum levels and positivity of IL-6 and CA 125 in ovarian cancer in relation to different clinicopathological parameters

| | No. of sera tested | IL-6 Median (range) (pg ml$^{-1}$) | IL-6 >6 pg ml$^{-1}$ No. (%) | CA 125 >35 U ml$^{-1}$ No. (%) |
|---|---|---|---|---|
| Total | 114 | 5.8 (0.3-660) | 57 (50) | 99 (87) |
| Stage | | | | |
| I | 19 | 1.5 (0.3-20) | 7 (37) | 10 (53) |
| II | 8 | 5 (0.4-90) | 3 (37) | 7 (87) |
| III | 62 | 6.9 (0.3-660) | 33 (53) | 57 (92) |
| IV | 25 | 7 (0.7-180) | 14 (56) | 25 (100) |
| Histology | | | | |
| Serous | 80 | 7 (0.3-660) | 43 (54) | 72 (90) |
| Mucinous | 9 | 1.5 (0.4-55) | 2 (22) | 7 (78) |
| Endometrioid | 6 | 4.4 (0.4-20) | 2 (33) | 5 (83) |
| Undifferentiated | 8 | 4.5 (0.4-26) | 4 (50) | 8 (100) |
| Others | 11 | 7.7 (0.4-55) | 6 (54) | 7 (64) |
| Grading | | | | |
| 1 | 14 | 9.8 (0.4-120) | 7 (50) | 11 (78) |
| 2 | 30 | 4.3 (0.3-110) | 11 (37) | 24 (80) |
| 3 | 62 | 7 (0.3-660) | 35 (56) | 56 (90) |
| Ascites[a] | | | | |
| No | 41 | 4.9 (0.3-660) | 20 (49) | 37 (90) |
| Yes | 46 | 8.9 (0.4-280) | 27 (59) | 44 (96) |
| Residual tumour after surgery[a] | | | | |
| ≤2 cm | 48 | 4.4 (0.3-660) | 21 (44)* | 45 (94) |
| >2 cm | 39 | 8.3 (0.4-280) | 26 (67) | 36 (92) |

[a]Only patients with stage III and IV disease are considered. *$P = 0.02$.

**Figure 1** **a**, Overall survival in patients with stage II, III or IV ovarian cancer in relation to IL-6 serum levels. **b**, Overall survival in patients with stage II, III or IV ovarian cancer with complete and partial response to chemotherapy in relation to IL-6 serum levels.

the overall survival of patients with stage II, III or IV ovarian cancer with a complete or partial response to chemotherapy. In this case there was also a statistically significant difference between patients with high IL-6 levels and a poor prognosis and those with low IL-6 and a better prognosis ($P = 0.0227$). Univariate analysis (Table II) showed that stage IV disease, post-operative residual tumour diameter greater than 2 cm, grading and high pretreatment IL-6 serum levels were significantly correlated with a shortened survival. Multivariate analysis revealed that only FIGO stage and pretreatment IL-6 levels were significantly correlated with a high risk of death.

**Table II** Univariate and multivariate analysis of survival in stage II, III and IV patients

| | Median survival (months) | Univariate P-value | Multivariate P-value |
|---|---|---|---|
| FIGO stage | | | |
| II-III | 28 | 0.0169 | 0.0023 |
| IV | 17 | | |
| Residual tumour after surgery | | | |
| ≤2 cm | 51 | 0.007 | 0.7702 |
| >2 cm | 20 | | |
| Grading | | | |
| 1-2 | 38 | 0.038 | 0.15 |
| 3 | 22 | | |
| Pretreatment IL-6 | | | |
| <6 pg ml$^{-1}$ | 51 | 0.009 | 0.0081 |
| ≥6 pg ml$^{-1}$ | 21 | | |

### Discussion

Prognostic characterisation of patients with advanced ovarian cancer is still inadequate. Therefore, identification of variables correlating with tumour aggressiveness would contribute to the selection of therapy for individual patients.

Our results show that elevated IL-6 serum levels in patients with primary epithelial ovarian cancer correlate with poor prognosis. Multivariate analysis showed that the association of high IL-6 levels with poor survival was independent of the other known prognostic factors such as stage and residual disease.

These results are consistent with previous data reported by Reibnegger *et al.* (1992) showing an association between IL-6 and disease progress in multiple myeloma. High serum IL-6 is also an adverse prognostic factor in renal cancer (Blay *et al.*, 1992) and glioblastoma (Van Meir *et al.*, 1990). Several observations support the hypothesis that IL-6 expression is related to tumour aggressiveness: (i) IL-6 is a growth factor for ovarian and renal carcinoma cells (Miki *et al.*, 1989; Wu *et al.*, 1992); (ii) renal cancer cells express IL-6 receptor mRNA (Miki *et al.*, 1989), suggesting that this cytokine may function in an autocrine fashion; and (iii) Tamm *et al.* (1989) showed that exogenous IL-6 increases the motility and decreases adherence of the breast carcinoma cell lines T47D and ZR75-1, suggesting that *in vivo* IL-6 may promote tumour metastasis and invasiveness. Alternatively, IL-6 could exert an adverse effect through a modulation of the anti-tumour response. Although *in vitro* IL-6 enhances the functions of cytotoxic T lymphocytes and natural killer cells and synergises with IL-2 to increase the cytotoxic activity of peripheral blood T lymphocytes (Okada *et al.*, 1988), high concentrations of IL-6 inhibit the immune tumour response in a murine model (Tanner and Tosado, 1991). In view of the high concentration of IL-6 found in ascites of ovarian cancer patients, this cytokine may play a similar role in this disease (Watson *et al.*, 1990).

In our series, increased IL-6 serum levels were accompanied by augmented IL-6R concentration. The regulation *in vivo* of the shedding of soluble IL-6Rs, the function and the significance of these soluble receptors in biological fluids are not currently understood. It has been suggested, however, that pathological conditions involving elevated levels of IL-6 might also be associated with increased production of soluble IL-6Rs (Honda *et al.*, 1992). Accordingly, ovarian cancer patients exhibited increased serum levels of both IL-6 and soluble IL-6Rs.

The present report on a larger population confirms our previous finding that IL-6 is not a useful tumour marker for ovarian cancer. The sensitivity of IL-6 is lower than that of CA 125. Furthermore, combined IL-6 and CA 125 only slightly increased the overall sensitivity as compared with CA 125 alone.

This study, together with the *in vitro* results indicating that ovarian cancer cell proliferation and tumour angiogenesis are affected by IL-6 (Motro *et al.*, 1990), suggests new therapeutic strategies based on IL-6 interference by antisense oligonucleotides or monoclonal antibodies. In this regard, recent reports have shown that addition in culture of anti-IL-6 antibody and/or IL-6 mRNA antisense oligonucleotides inhibits the proliferation of carcinoma cell lines (Miki *et al.*, 1989; Watson *et al.*, 1993).

In conclusion, data from the present study suggest that the assessment of serum IL-6 at the time of initial surgery may allow the identification of a subset of patients with a particularly poor prognosis. This aspect should be investigated in a larger clinical trial including other biological parameters in the multivariate analysis.

**Acknowledgements**
This work was partially supported by a grant from CNR, finalised Project No. 94.01209.PF39.

**References**

BENEDETTI PANICI P, GREGGI S, SCAMBIA G, BAIOCCHI G, LO MONACO M, CONTI G AND MANCUSO S. (1993). Efficacy and toxicity of very high-dose cisplatin in advanced ovarian carcinoma: 4-year survival analysis and neurological follow-up. *Int. J. Gynecol. Cancer*, **3**, 44-53.

BEREK JS, CHUNG CBS, KALDI KBS, WATSON JM, KNOX RM AND MARTINEZ-MAZA O. (1991). Serum interleukin-6 levels correlate with disease status in patients with epithelial ovarian cancer. *Am. J. Obstet. Gynecol.*, **164**, 1038-1043.

BLAY JY, NEGRIER S, COMBARET V, ATTALI S, GOILLIOT E, MERROUCHE Y, MERCATELLO A, RAVAULT A, TOURANI JM, MOSKOVTCHENKO JF, PHILIP T AND FAVROT M. (1992). Serum level of interleukin-6 as a prognosis factor of metastatic renal carcinoma. *Cancer Res.*, **52**, 3317-3322.

DIXON WS. (1981). *BMDP Statistical Software*. University of California Press: Berkeley, CA.

HONDA M, YAMAMOTO S, CHENG M, YASUKAWA K, SRUKI H, SAITO T, OSUGI Y, TOKUNAGA T AND KISHIMOTO T. (1992). Human soluble IL-6 receptor: its detection and enhanced released by HIV infection. *J. Immunol.*, **148**, 2175-2189.

KAPLAN E AND MEIER P. (1958). Non parametric estimation from incomplete observation. *J. Am. Stat. Assoc.*, **53**, 457-481.

MANTEL N. (1966). Evaluation of survival data and two new rank order statistics arising in its consideration. *Cancer Chemother. Rep.*, **50**, 163-170.

MIKI S, IWANO M, MIKI Y, YAMAMOTO M, TANG B, YOKOKAWA K, SONODA T, HIRANO T AND KISHIMOTO T. (1989). Interleukin-6 (IL-6) functions as in vitro autocrine growth factor in renal cell carcinomas. *FEBS Lett.*, **250**, 607-610.

MOTRO B, ITIN A, SACHS L AND KESHET E. (1990). Pattern of interleukin-6 gene expression in vivo suggests a role for this cytokine in angiogenesis. *Proc. Natl Acad. Sci. USA*, **87**, 3092-3096.

OKADA M, KITAHARA M, KISHIMOTO S, MATSUDA T, HIRANO T AND KISHIMOTO T. (1988). IL-6/BSF-2 functions as a killer helper factor in the *in vitro* induction of cytotoxic T-cells. *J. Immunol.*, **141(5)**, 1543-1549.

REIBNEGGER G, KRAINER M, HEROLD M, LUDWIG H, WATCHER H AND HUBER H. (1992). Predictive value of interleukin-6 and neopterin in patients with multiple myeloma. *Cancer Res.*, **54**, 3317-3322.

SCAMBIA G, BENEDETTI PANICI P, PERRONE L, SONSINI C, GIANNELLI S, GALLO A, NATALI PG AND MANCUSO S. (1990). Serum levels of tumor associated glycoprotein (TAG 72) in patients with gynecological malignancies. *Br. J. Cancer*, **62**, 147-151.

SCAMBIA G, TESTA U, BENEDETTI PANICI P, MARTUCCI R, FOTI E, PETRINI M, AMOROSO M, MASCIULLO V, PESCHLE C AND M MANCUSO S. (1994). Interleukin-6 serum levels in patients with gynecological tumors. *Int. J. Cancer*, **57**, 318-323.

SEROV SF AND SCULLY RE. (1973). Histological typing of ovarian tumors. In *International Histological Classification of Tumors*, No. 9. World Health Organization: Geneva.

TAMM I, CARDINALE I, KRUEGER J, MURPHY JS, MAY LT AND SEGHALL PB. (1989). Interleukin-6 decreases cell-cell association and increases motility of ductal breast carcinoma cells. *J. Exp. Med.*, **170**, 1649-1669.

TANNER J AND TOSATO G. (1992). Impairment of natural killer functions by interleukin-6 increases lymphoblastoid cell tumorigenicity in athymic mice. *J. Clin. Invest.*, **88**, 239-247.

VAN MEIR E, SAWAMURA Y, DISERENS AC, HAMOU MF AND DE TRIBOLET N. (1990). Human glioblastoma cells release interleukin-6 in vivo and in vitro. *Cancer Res.*, **50**, 6683-6688.

WATSON JM, SENSITAFFAR JL, BEREK JS AND MARTINEZ-MAZA O. (1990). Constitutive production of interleukin-6 by ovarian cancer cell lines and by primary ovarian tumor cultures. *Cancer Res.*, **50**, 6959-6965.

WATSON JM, BEREK JS AND MARTINEZ-MAZA O. (1993). Growth inhibition of ovarian cancer cells induced by antisense IL-6 oligonucleotides. *Gynecol. Oncol.*, **49**, 8-15.

WU S, RODABAUGH K, MARTINEZ-MAZA O, WATSON JM, SILVERSTEIN DS, BOYER CM, PETERS WP, WEINBERG B, BEREK JS AND BAST Jr, RC. (1992). Stimulation of ovarian tumor cell proliferation with monocytes products including interleukin-1, interleukin-6, and tumor necrosis factor-A. *Am. J. Obstet. Gynecol.*, **166**, 997-1007.


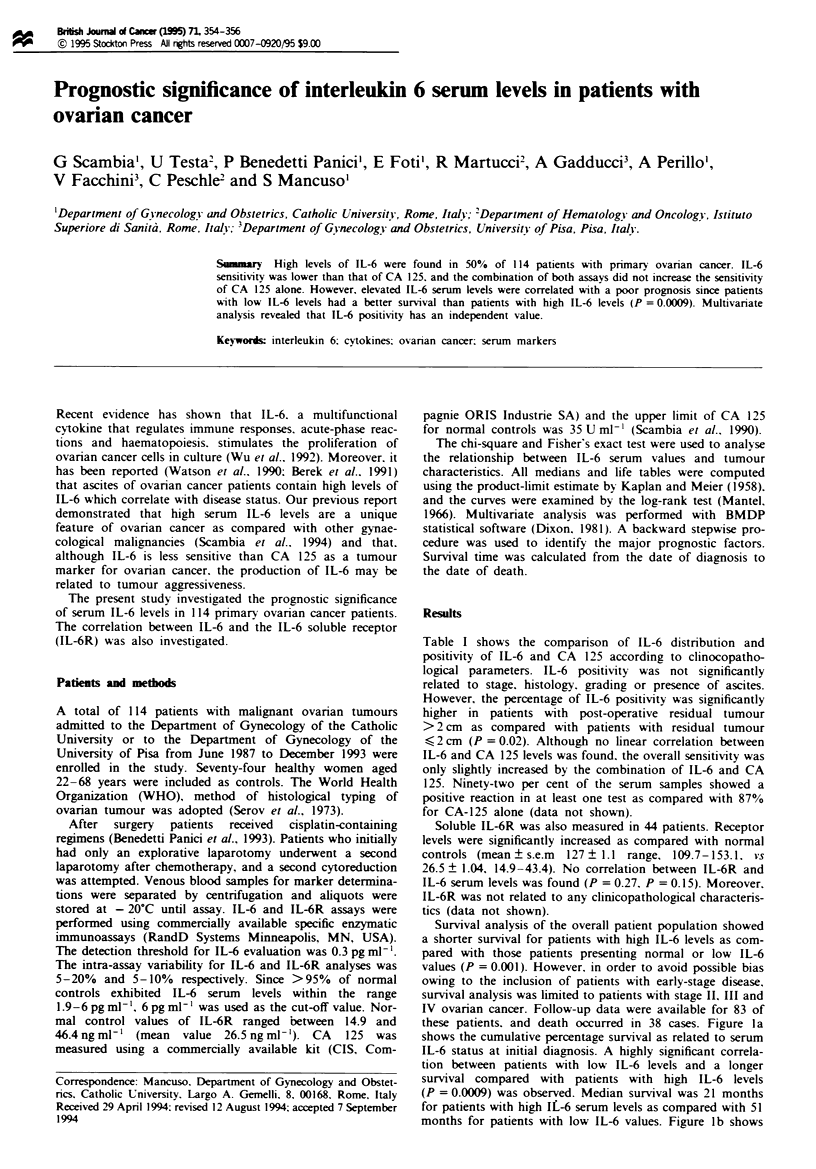

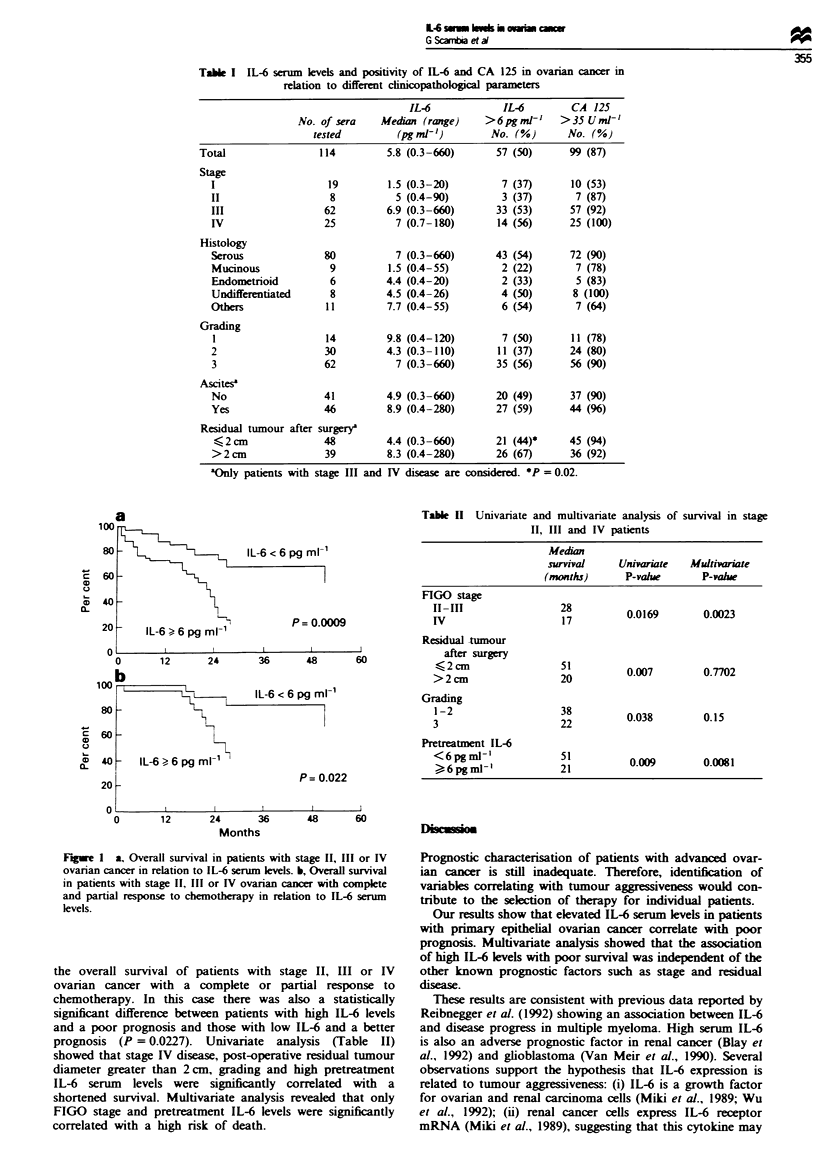

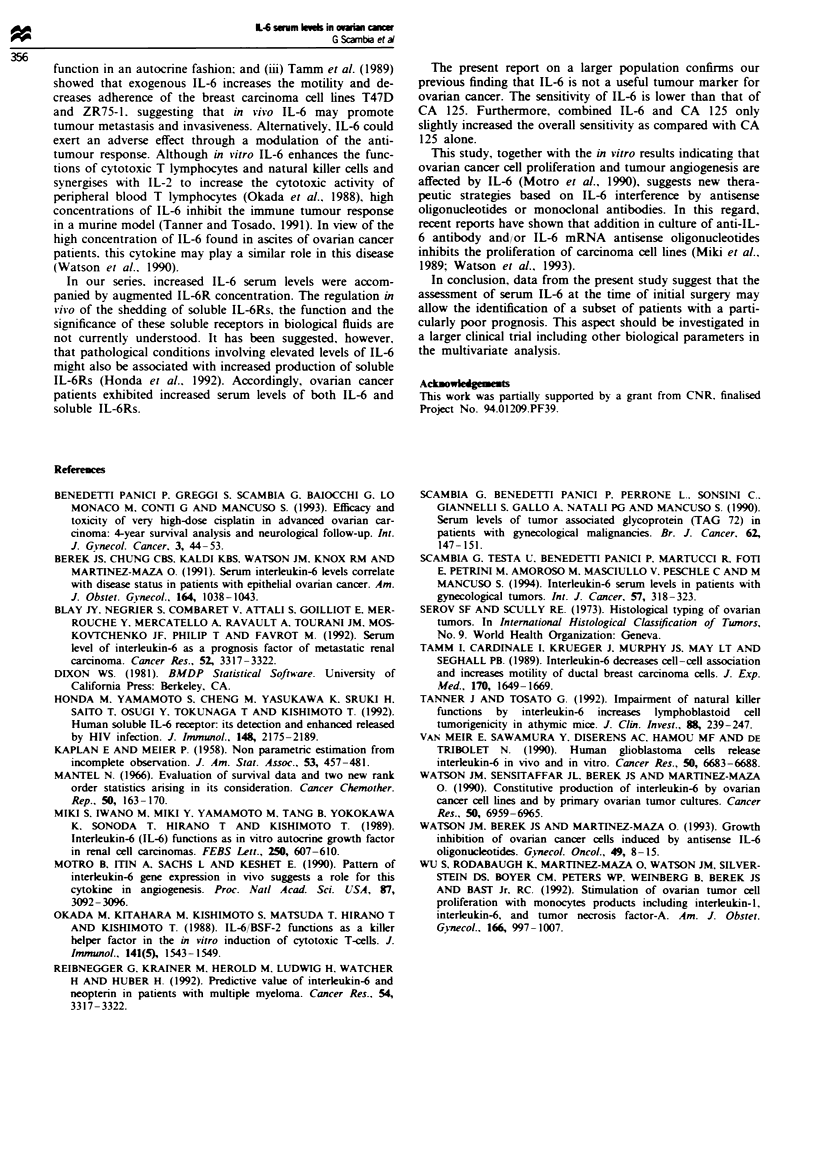


## References

[OCR_00325] Benedetti Panici P., Greggi S., Scambia G., Baiocchi G., Lomonaco M., Conti G., Mancuso S. (1993). Efficacy and toxicity of very high-dose cisplatin in advanced ovarian carcinoma: 4-year survival analysis and neurological follow-up.. Int J Gynecol Cancer.

[OCR_00332] Berek J. S., Chung C., Kaldi K., Watson J. M., Knox R. M., Martínez-Maza O. (1991). Serum interleukin-6 levels correlate with disease status in patients with epithelial ovarian cancer.. Am J Obstet Gynecol.

[OCR_00338] Blay J. Y., Negrier S., Combaret V., Attali S., Goillot E., Merrouche Y., Mercatello A., Ravault A., Tourani J. M., Moskovtchenko J. F. (1992). Serum level of interleukin 6 as a prognosis factor in metastatic renal cell carcinoma.. Cancer Res.

[OCR_00349] Honda M., Yamamoto S., Cheng M., Yasukawa K., Suzuki H., Saito T., Osugi Y., Tokunaga T., Kishimoto T. (1992). Human soluble IL-6 receptor: its detection and enhanced release by HIV infection.. J Immunol.

[OCR_00358] Mantel N. (1966). Evaluation of survival data and two new rank order statistics arising in its consideration.. Cancer Chemother Rep.

[OCR_00363] Miki S., Iwano M., Miki Y., Yamamoto M., Tang B., Yokokawa K., Sonoda T., Hirano T., Kishimoto T. (1989). Interleukin-6 (IL-6) functions as an in vitro autocrine growth factor in renal cell carcinomas.. FEBS Lett.

[OCR_00369] Motro B., Itin A., Sachs L., Keshet E. (1990). Pattern of interleukin 6 gene expression in vivo suggests a role for this cytokine in angiogenesis.. Proc Natl Acad Sci U S A.

[OCR_00376] Okada M., Kitahara M., Kishimoto S., Matsuda T., Hirano T., Kishimoto T. (1988). IL-6/BSF-2 functions as a killer helper factor in the in vitro induction of cytotoxic T cells.. J Immunol.

[OCR_00387] Scambia G., Benedetti Panici P., Perrone L., Sonsini C., Giannelli S., Gallo A., Natali P. G., Mancuso S. (1990). Serum levels of tumour associated glycoprotein (TAG 72) in patients with gynaecological malignancies.. Br J Cancer.

[OCR_00395] Scambia G., Testa U., Panici P. B., Martucci R., Foti E., Petrini M., Amoroso M., Masciullo V., Peschle C., Mancuso S. (1994). Interleukin-6 serum levels in patients with gynecological tumors.. Int J Cancer.

[OCR_00406] Tamm I., Cardinale I., Krueger J., Murphy J. S., May L. T., Sehgal P. B. (1989). Interleukin 6 decreases cell-cell association and increases motility of ductal breast carcinoma cells.. J Exp Med.

[OCR_00411] Tanner J., Tosato G. (1991). Impairment of natural killer functions by interleukin 6 increases lymphoblastoid cell tumorigenicity in athymic mice.. J Clin Invest.

[OCR_00417] Van Meir E., Sawamura Y., Diserens A. C., Hamou M. F., de Tribolet N. (1990). Human glioblastoma cells release interleukin 6 in vivo and in vitro.. Cancer Res.

[OCR_00424] Watson J. M., Berek J. S., Martínez-Maza O. (1993). Growth inhibition of ovarian cancer cells induced by antisense IL-6 oligonucleotides.. Gynecol Oncol.

[OCR_00420] Watson J. M., Sensintaffar J. L., Berek J. S., Martínez-Maza O. (1990). Constitutive production of interleukin 6 by ovarian cancer cell lines and by primary ovarian tumor cultures.. Cancer Res.

[OCR_00429] Wu S., Rodabaugh K., Martinez-Maza O., Watson J. M., Silberstein D. S., Boyer C. M., Peters W. P., Weinberg J. B., Berek J. S., Bast R. C. (1992). Stimulation of ovarian tumor cell proliferation with monocyte products including interleukin-1, interleukin-6, and tumor necrosis factor-alpha.. Am J Obstet Gynecol.

